# Cell Contact with Endothelial Cells Favors the In Vitro Maintenance of Human Chronic Myeloid Leukemia Stem and Progenitor Cells

**DOI:** 10.3390/ijms231810326

**Published:** 2022-09-07

**Authors:** Patricia Torres-Barrera, Dafne Moreno-Lorenzana, José Antonio Alvarado-Moreno, Elena García-Ruiz, Cesar Lagunas, Hector Mayani, Antonieta Chávez-González

**Affiliations:** 1 Laboratorio de Células Troncales Leucémicas, Unidad de Investigación Médica en Enfermedades Oncológicas, CMN Siglo XXI, Instituto Mexicano del Seguro Social, CDMX 06725, Mexico; 2 Posgrado en Ciencias Biológicas, UNAM, CDMX 04510, Mexico; 3 CONACYT-Instituto Nacional de Pediatria, CDMX 04530, Mexico; 4 Unidad de Investigación Médica en Trombosis Hemostasia y Aterogenesis, Instituto Mexicano del Seguro Social, CDMX 03100, Mexico; 5 Departamento de Hematología, Hospital de Especialidades, CMN La Raza, Instituto Mexicano del Seguro Social, CDMX 02990, Mexico; 6 Departamento de Cirugías de Cadera, Hospital General “Villa Coapa” Instituto Mexicano del Seguro Social, CDMX 14310, Mexico; 7 Laboratorio de Células Troncales Hematopoyéticas, Unidad de Investigación Médica en Enfermedades Oncológicas, CMN Siglo XXI, Instituto Mexicano del Seguro Social, CDMX 06725, Mexico

**Keywords:** Leukemic Stem Cells, CML, Microenvironment, Endothelial Cells

## Abstract

Chronic Myeloid Leukemia (CML) originates in a leukemic stem cell that resides in the bone marrow microenvironment, where they coexist with cellular and non-cellular elements. The vascular microenvironment has been identified as an important element in CML development since an increase in the vascularization has been suggested to be related with poor prognosis; also, using murine models, it has been reported that bone marrow endothelium can regulate the quiescence and proliferation of leukemic stem and progenitor cells. This observation, however, has not been evaluated in primary human cells. In this report, we used a co-culture of primitive (progenitor and stem) CML cells with endothelial colony forming cells (ECFC) as an in vitro model to evaluate the effects of the vascular microenvironment in the leukemic hematopoiesis. Our results show that this interaction allows the in vitro maintenance of primitive CML cells through an inflammatory microenvironment able to regulate the proliferation of progenitor cells and the permanence in a quiescent state of leukemic stem cells.

## 1. Introduction

Chronic Myeloid Leukemia (CML) is a myeloproliferative disease characterized by high numbers of myeloid cells in bone marrow (BM) and peripheral blood. The origin, maintenance and development of CML majorly depend on the presence of reciprocal translocation t (9:22) (q34; q11) [1]. The resultant BCR::ABL1 oncoprotein has a constitutive tyrosine kinase activity, which is involved in several cell abnormalities, such as proliferation and cell adhesion [2,3]. CML originates in a leukemic stem cell (LSC; CD34+CD38-lin-cells) population [4]. LSCs reside within the bone marrow microenvironment (BMM) where they coexist with normal hematopoietic stem cells (HSCs). During disease progression, HSCs are displaced by LSCs and their progeny. Indeed, it has been shown that leukemic cells modify their surrounding niche, e.g., by inducing a pro-inflammatory environment [5,6], generating a permissive space for LSC self-renewal, differentiation and survival [7].

The vascular microenvironment has been identified as an important element in CML development. Increased BM vascularization after the malignancy establishment has been observed in murine models [8,9], as well as in BM from CML patients, where an association with poor prognosis was suggested [8]. In murine models of CML it has been reported that the BM endothelium can regulate the quiescence [10,11] and proliferation [12] of leukemic stem and progenitor cells. This observation, however, has not been evaluated in primary human cells.

In the present report, we describe the co-culture of primitive leukemic cells with endothelial colony forming cells (ECFC) [13], as an in vitro model for the interaction of leukemic cells and a vascular microenvironment, in order to analyze the effect of the endothelium on the maintenance of stem and progenitor cells from CML patients.

## 2. Results

### 2.1. Co-Culture with Endothelial Cells Allows Maintenance of Primitive CML Cells

**Figure 1:**
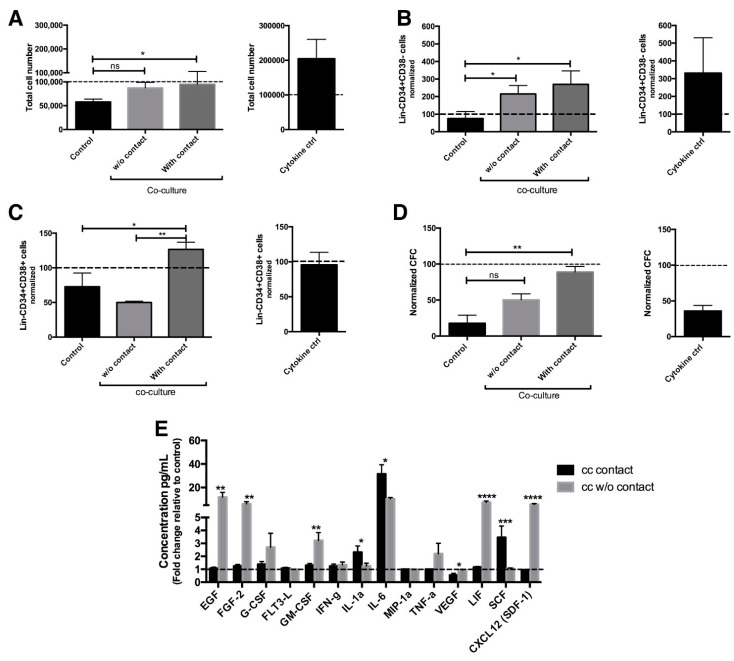
Co-culture with endothelial cells allows the CML primitive cells’ maintenance. A hundred thousand Lin-CD34+ cells from CML patients were co-cultured with or without contact with EC for 3 days in absence of any cytokine supplement. Basal culture medium (control) or StemSpan medium supplemented with cytokines (cytokine ctrl) were used as negative and positive controls, respectively. (**A**) Total CML cell number was evaluated after three days in EC co-cultures. The cells were counted with Trypan blue staining and non-viable cells were excluded. (**B**) Stem (Lin-CD34+CD38-) or (**C**) progenitor (Lin-CD34+CD38+) cell number was evaluated after 3 days of co-culture. All data were normalized to the initial cell numbers in individual cell subsets obtained (post-enrichment and before culture). (**D**) After three days of co-culture, 3000 hematopoietic cells were sub-cultured in methyhlcellulose for 14 days and CFC was evaluated. The number of colonies was normalized with respect to CFC at zero time from each sample used. (**E**) Cytokines’ detection was performed by multiplexing analyte assay. Co-cultured cytokine concentration was normalized with the cytokine concentration in control after three days in culture. The data are expressed as mean ± SEM of 3–5 independent experiments in triplicate. * *p* ≤ 0.05, ** *p* ≤ 0.005, *** *p* ≤ 0.0005, **** *p* < 0.0001.

Based on the fact that the vascular microenvironment is able to regulate murine malignant hematopoiesis and considering that there is no information available in humans, we set up an in vitro model in which human endothelial cells (EC) were co-cultured with a cell population enriched in stem and progenitor (considered as primitive cells) CML cells. As a first approach, Lin-CD34+ cells, obtained from untreated CML patients, were cultured for 3 days with or without direct contact with EC derived from healthy donors. The cultures were performed in a basal medium without serum and in the absence of any supplemental cytokines, so that specific effects of the EC could be assessed with the minimal influence of exogenous factors. As shown in Figure 1a, the co-culture of CML cells with EC had a maintenance effect on CML cells. This effect was not observed in the absence of EC (control). It is noteworthy that the total CML population doubled when cells were cultured in the absence of endothelial cells but in the presence of hematopoietic stimulatory cytokines (cytokine ctrl).

Co-culture of endothelial cells with CML cells, without or with contact, resulted in a 110 and 170% increase in the levels of Lin-CD34+CD38- cells, respectively, as compared to the initial cell number (Figure 1b). The latter was similar to the increase seen in the cytokine control. As expected, in control cultures (basal medium only) there was a decrease in the number of Lin-CD34+CD38- cells (Figure 1b and Figure S1a). In the case of Lin-CD34+CD38+ cells, a 20% increase was observed when they were in contact with EC. Such an increase did not occur in the cytokine control cultures and was statistically significant when compared to the negative control and to co-cultures without contact, where the population was reduced by half (Figure 1c).

In order to determine the effect of co-cultures on the levels of colony forming cells (CFC), colony assays were performed. Figure 1d shows that when leukemic cells were in contact with EC, the levels of CFC were maintained; in contrast, in co-cultures without contact, CFC levels were reduced by 50%. CFC numbers were reduced by 65% and 83% in the cytokine control and basal medium only control, respectively (Figure 1d). In addition, in co-cultures without contact, the majority of the colonies observed corresponded to myeloid (CFU-G and CFU-M) and late erythroid (CFU-E) colonies, whereas, in contact co-cultures, a high proportion of BFU-E, CFU GM, and CFU-Mix colonies was observed, suggesting that direct contact favored the presence of more primitive progenitors (Figure S1b).

To identify soluble factors that could be involved in the maintenance of leukemic cells in culture, we performed a multiplex analysis in the supernatants after 3 days of culture. In co-cultures without contact, we found a high production of inflammatory cytokines and soluble molecules related to myeloid differentiation. On the other hand, in cultures with direct contact, the secretion of inflammatory molecules was reduced, and only high levels of IL-1a, IL-6 and SCF were observed (Figure 1e). It is noteworthy that all of these cytokines are involved in CML cell maintenance [5,14]. Taken together, these data indicate that contact with endothelial cells favors the maintenance of CML primitive cells in culture.

### 2.2. Leukemic Stem Cells Remain Attached to Endothelial Cells

**Figure 2:**
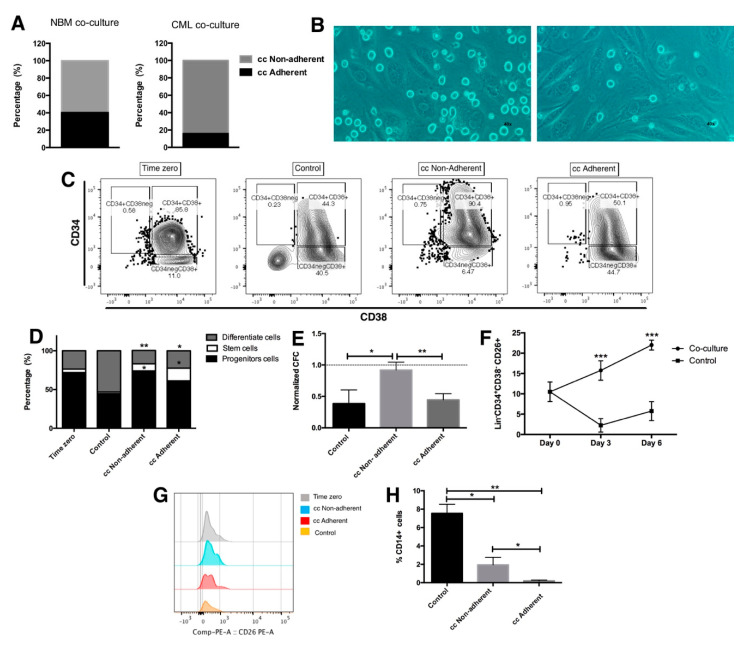
CML primitive cells had different affinity to endothelial microenvironment. Lin-CD34+ cells from CML patients were co-cultured in contact with EC for three days in absence of any cytokines to identify CML sub-fractions. (**A**) Percentage of adherent and non-adherent hematopoietic fractions in CML and NBM contact co-cultures. (**B**) Representative photographs of a co-culture with both adherent (right) and non-adherent (left) cell fraction. (**C**) Representative dot plot and (**D**) percentage of different CML populations (differentiate Lin+CD34-; progenitor CD34+CD38+ and stem CD34+CD38- cells) at the beginning of the experiment (zero time) and after three days in the absence (control) or presence of contact with EC where the adherent and non-adherent fraction were analyzed individually. (**E**) After three days of co-culture, 3,000 hematopoietic cells were sub-cultured in methyhlcellulose for 14 days and CFC was evaluated. The number of colonies was normalized with respect to the initial CFC in each sample used at the beginning of the experiment. (**F**) CML stem cells CD26+ (Lin-CD34+CD38-CD26+) were evaluated after 3 and 6 days of co-culture in a total population as well and (**G**) in adherent and non-adherent fractions. (**H**) The percentage of CD14+ myeloid cells were also determinate. The data are expressed as mean ± SEM (**E**,**F**,**H**) or mean (**A**,**D**) of 3–4 independent experiments by triplicate. * *p* ≤ 0.05, ** *p* ≤ 0.005, *** *p* ≤ 0.0005.

Based on the results presented above, indicating the importance of direct contact between endothelial cells and CML primitive cells, we focused on the direct contact co-cultures. We observed that, after allowing the interaction between both cell types for 3 days, two different fractions of hematopoietic cells were detected: a fraction consisting of hematopoietic cells directly attached to the endothelium (cc adherent), representing 15.9% of the total cells, and a second fraction that remained in the supernatant of the co-culture (cc non-adherent), representing 84% of the cells (Figure 2a,b). Interestingly, these proportions were considerably different in cultures of normal hematopoietic cells (Figure 2a), in which there was a higher number of cells attached to the endothelium (40.2%).

When the cell immunophenotype was analyzed in each one of the co-cultured fractions, we detected stem, progenitor and mature cells at different frequencies (Figure 2c,d). Within the adherent fraction, 16%, 61% and 22% showed stem, progenitor and mature cell immunophenotypes, respectively. In the non-adherent fraction, the percentages of cells with stem, progenitor and mature cell immunophenotypes were 9%, 74% and 17%, respectively. It is noteworthy that the increase in Lin-CD34+CD38+ cells correlated with the number of CFC evaluated (Figure 2e).

Considering that CD26 has been proposed as a specific marker of CML stem cells [15], its expression was evaluated in the total hematopoietic cells present in co-cultures, as well as in the adherent and non-adherent sub-fractions of direct contact co-cultures. Figure 2f shows a 50% and 120% increase in the levels of lin-CD34+CD38-CD26+ cells after 3 and 6 days in the presence of EC, respectively. Interestingly, in the control cultures, such a population was decreased. In keeping with this result, the expression of CD26 was detected after 3 days in both attached and non-attached cells to endothelia (Figure 2g), while the number of mature (CD34-Lin+; Figure 2d) and myeloid (CD14+; Figure 2h) cells was decreased.

Similar results were observed when the MEG-01 CML cell line was used. Indeed, the stem cell population was preferentially enriched in the adherent fraction (from 0.25% at zero time to 1.34% after 3 days of co-culture). An increase in CD26 expression was also observed in this population, with a concomitant reduction in the differentiation markers CD11b+, CD61 and CD41 (Figure S2). These results suggest that in our culture system, differentiated and non-differentiated CML cells may have different affinities to endothelial cells.

### 2.3. The Proliferation Status of CML LSC Is Regulated by the Co-Culture with EC

In order to determine the role of EC in CML proliferation and retention of the stem cell immunophenotype, the CML bulk (CD45+) and stem cell (CD34+CD38-) populations were tracked at 24, 48 and 72 h by multi-staining with CFSE, and primitive immunophenotype markers after 3 days of co-culture in the presence of EC.

**Figure 3:**
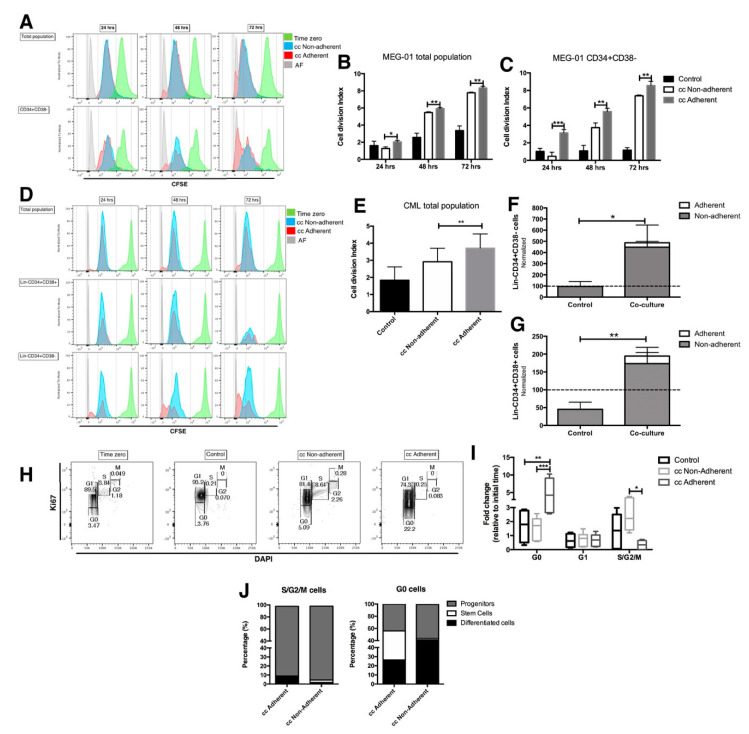
The endothelial microenvironment could induce proliferation and cell arrest in CML stem cells: Cells from CML were co-cultured in contact with EC for three days in absence of any cytokines to evaluate proliferation at different times (using CFSE staining) or cell cycle staining after three days (through Ki67 and DAPI incorporation). (**A**) Representative histograms of CFSE assay at 24, 48 and 72 h of culture in total or stem cell populations from MEG-01 cell line or (**D**) primary CML sample. Cell division index in (**B**) bulk and (**C**) stem cell population (CD34+CD38-) of MEG-01 cell line at 24, 48 and 72 h of co-culture. (**E**) Primary CML total cells after three days of co-culture. Number of cells with (**F**) stem (Lin-CD34+CD38-) and (**G**) progenitor (Lin-CD34+CD38+) immunophenotype after 72 h of co-culture. Cell number was normalized with respect to the initial number of progenitor and stem cells in each sample used. (**H**) Representative dot plots of one CML sample after three days in the different co-culture conditions. (**I**) Fold change of cell cycle phases, relative to time zero of each sample used. (**J**) Percentage of different hematopoietic subpopulations in each co-culture condition and cell cycle phase. The data are expressed as mean ± SEM or only the mean of 3–6 independent experiments in triplicate. * *p* ≤ 0.05, ** *p* ≤ 0.005, *** *p* ≤ 0.0005.

Figure 3 shows that in the total CML population in the MEG-01 cell line, the fraction that remains attached to EC has the highest proliferation potential (Figure 3a, red histogram), which correlates with a significant increase in the cell division index (Figure 3b). The same result was observed in the total population from the primary CML cells (Figure 3d, red histogram and 3e), but it was not detected in normal bone marrow cells where adherent and non-adherent cells showed a similar proliferation and also had a similar cell division index (Figure S3a,b).

When the stem cell populations (CD34+CD38-) from the MEG-01 cell line and from one CML primary sample (lin-CD34+CD38-) were tracked, more cell divisions were observed in the fraction attached to the endothelial layer in relation to non-adherent cells (Figure 3a,d). This proliferation ability was more evident than was previously described for the total CML cells. Consistent with this result, the cell division index was higher in the adherent than in the non-adherent stem cell-containing fraction of MEG-01 cells (Figure 3c). Moreover, in the sample of CML primary cells that remained adherent to the EC, the total number of cells with the stem and progenitor immunophenotype was increased 4- and 1.7-fold with respect to the initial number in the CML control (Figure 3f,g). A similar result was obtained in stem cells from the MEG-01 cell line (Figure S3c). All these results suggest a special ability of leukemic stem cells to remain and proliferate in the endothelial microenvironment even if they are found in the adherent fraction.

To verify the proliferative ability of Lin-CD34+CD38- in contact with EC, a cell cycle status was evaluated at the end of each co-culture. The results show that after 72 h in contact with EC, there was a 5-fold increase in adherent cells in the G0 phase with respect to their number at time zero, while the S/G2/M phases were reduced. In contrast, cells in the non-adherent fraction had an increase in the proliferative phases (S/G2/M) (Figure 3h,i). In cultures of MEG-01 cells, an increase in the G0 phase and a reduction in the S/G2/M phases in EC-attached cells were also observed (Figure S3f). These results differ from those of normal bone marrow cells, in which the quiescent population remained similar before and after co-culture (Figure S3d,e), suggesting that this behavior is exclusive to CML cells, and that it depends on cell contact.

Interestingly, 29.5% of the cells in G0, and that were attached to EC, showed a stem cell phenotype; in contrast, only 2.7% of G0 cells in the non-attached fraction showed a stem cell phenotype (Figure 3j). Moreover, cells with a stem cell phenotype were not observed in the active cell cycle phases (S/G2/M), where the highest percentage (90%) corresponded to progenitor cells (Figure 3j).

All these results suggest that the endothelial microenvironment could induce proliferation and/or quiescence in CML stem cells and this could be related to the type of interactions that are established between both cell linages.

### 2.4. The Cytokine Profile and Notch Expression Are Modified after Co-Culture with EC

**Figure 4:**
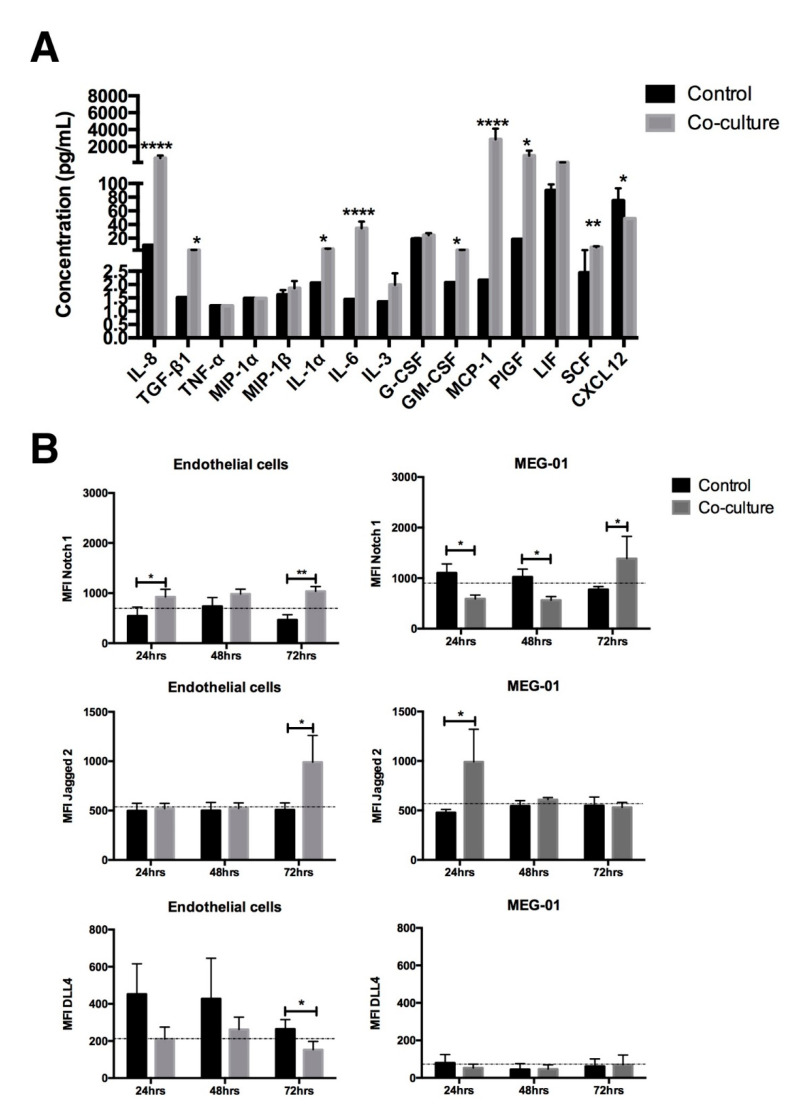
Cytokine profile and notch expression after co-culture with EC: (**A**) cytokine concentration (pg/mL) in co-culture supernatants was evaluated (by milliplex immunoassay) after three days of co-culture. (**B**) Notch1, Jagged-2 and DLL4 expression in endothelial cells and MEG-01 cell line after 24, 48 and 72 h of co-culture were analyzed by flow cytometry. The data are expressed as mean ± SEM or only the mean of 3 independent experiments in triplicate; * *p* ≤ 0.05, ** *p* ≤ 0.005, **** *p* ≤ 0.0001.

In trying to explain the persistence of leukemic cells in the presence of the endothelial microenvironment, their cytokine profile was analyzed after 3 days of co-culture. Figure 4a shows that, as compared to control conditions (no contact co-culture), there was a significant increase in soluble factors, such as: IL-1a, IL-6 and PIGF that have been related to leukemic maintenance at the expense of normal hematopoiesis. Molecules that could trigger chronic inflammation and give rise to a pro-leukemic microenvironment, such as IL-8, TGF-b1, GM-CSF and MCP-1, were also considerably elevated. This scenario could be related with the proliferation observed, but could not necessarily explain the quiescence obtained.

Considering that in murine models adhesion molecules, such as Notch1 and their ligands, are involved in the maintenance of CML stem and progenitor cells [16], the expression of Notch1, Jagged 2 and DLL4 were analyzed in both co-culture components, the MEG-01 cell line and endothelial cells. As shown in Figure 4b, we found that after 72 h of co-culture, there was an increase in Notch-1 and Jagged-2 expression in leukemic and endothelial cells (Figure 4b). This time-point (72 h) correlated with the moment of leukemic cell arrest in the G0 phase of the cell cycle, and, although there are surely other molecules involved, the results of this study show that, in human CML, the endothelial microenvironment creates a suitable place for the maintenance of leukemic hematopoiesis, and that stem cell quiescence and proliferation, even in adherent conditions, could be associated with the communication that is established through direct contact and the soluble factor secretions, which contributes to leukemic permanence.

## 3. Discussion

Similar to normal hematopoiesis, the long-term CML persistence depends on the presence of the leukemic progenitor and stem cells. Cumulative evidence suggests that communication between the bone marrow microenvironment components and leukemic cells is essential for the development of disease [7]. In homeostasis, in vitro and in vivo models show that the endothelial cells provide key signals for the maintenance of healthy hematopoiesis, even under stress conditions, such as myeloablation [17]. In CML, recent studies with murine models have suggested that EC provide an essential microenvironment element that support the quiescence [10,11] and proliferation of leukemic primitive cells [12], both processes involved in the long-term maintenance of LSC. However, the role of EC in human CML hematopoiesis has not been described. In the present study, using an in vitro co-culture model with primary CML cells and ECFC, we showed that direct cell interaction provides a favorable microenvironment for the maintenance and expansion of CML stem and progenitor cells.

Our first results show that direct contact with EC favors the maintenance of the CML progenitor (CD34+CD38+lin-) and stem cells (CD34+CD38-lin-). When we analyzed the different localizations in direct contact co-cultures, we found that only 15% of CD34+ CML cells were attached to the endothelial layer, while 85% were found in the supernatant. This observation seems to be in keeping with previous reports that show that BCR::ABL1 tyrosine kinase promotes adhesion defects of the progenitor cells to the stromal microenvironment through crkL activation [18]. This is not observed in cultures of CD34+ cells from normal bone marrow (NBM), where we found 2.5 times more cells attached to the endothelium than in leukemic cultures (Figure 2a).

When tracking stem and progenitor CML cells after 3 days of co-culture, we found a higher percentage of cells with stem-like immunophenotypes attached to the endothelial layer in both the CML primary cells (Figure 2) and MEG-01 cell line (Figure S1). Although the leukemic stem subpopulation attached to the endothelium was arrested in phase G0 of the cell cycle at day 3 of co-culture, we observed that the same subpopulation had more cell divisions along 72 h of culture, as compared to the CML stem cells in the non-adherent fraction (Figure 3). A similar effect has been reported using human bone marrow mesenchymal stromal cells (MSC), which enhance the preservation of primitive leukemic cells, after 4 days in culture, and only stroma adhesion itself is sufficient for arresting the CML stem cells in the cell cycle, even in the presence of tyrosine kinase inhibitors and the low concentration of growth factors [19,20].

It is noteworthy that although in the adherent fraction there was a higher percentage of stem cells (25%) than in the non-adherent fraction (2%), the highest absolute number of CML stem cells was found in the non-adherent fraction of co-cultures (447 ± 98.7 vs. 39.6 ± 8.4 stem cells). Interestingly, the non-adherent leukemic stem cell subpopulation was in the S/G2/M phases of the cell cycle, after 3 days of co-culture. These results suggest that the CML stem cell population comprises two different subpopulations, one quiescent and able to remain attached to endothelial cells, and another with the ability to have multiple cell divisions; or that leukemic stem cells proliferate only when they are in the supernatant, as reported by Godavarthy and collaborators. Indeed, using a murine model, these authors showed that BCR::ABL1 is a negative regulator of CD44 necessary for cell adhesion between CML stem cells and bone marrow endothelium, thus causing a decrease in cell adhesion and enhancing CML stem cell proliferation [11]. In both cases, CML stem cells would be protected from their exhaustion. In the same line of evidence, Bhatia and co-workers reported that CML progenitor cells had less attachment to stromal cells compared to their normal counterparts, which confers proliferation advantage to the leukemic clone [21]. It is interesting that in the leukemic progenitor population there is a small proliferative fraction (S/G2/M) attached to the endothelial cells, which correlates with a previous report that showed that CML progenitors proliferate continuously, even when they are in contact with normal bone marrow stromal cells, evading negative regulation [22].

Maintenance of the CML leukemic cells through the secretion of soluble factors and adhesion molecules derived from BMM cells is a topic that has previously been addressed. However, the cytokine profile induced by contact between the CML primitive cells and ECFC has not previously been reported. In this work, we showed that in response to co-culture, there was the production of several molecules involved in the regulation of hematopoiesis, and that their concentration changed depending on whether there was direct contact, or not, between hematopoietic cells and ECFC. This, in turn, could result in the differentiation or maintenance of primitive hematopoietic cells.

In our culture conditions, we observed an increase in the levels of molecules related to an inflammatory microenvironment. In direct contact co-cultures, there was a significant increase in IL1 and IL6. Interestingly, these two cytokines have been shown to be involved in the maintenance of CML progenitor and stem cells [5,14]. The inflammatory cytokines produced by direct contact between CML CD34+ cells and normal EC have been proposed as pro-leukemic [6]. IL-8 and TGFβ have been reported as capable of modulating the permanence of leukemic cells [23,24]; PIGF promotes BM angiogenesis [9], and MCP1 has been shown to block the cell cycle of normal but not CML progenitor cells [25]. All of these cytokines were increased in our co-culture model.

After co-culture, CD26 expression was increased in CML stem cells. Such an increase may be involved in the decrease reported in CXCL12, the proteolytic target of CD26 [15], which could be relevant in the low cell adhesion observed in co-cultures with CD34+ CML cells. Considering that, under inflammatory conditions, BM endothelial cells can induce hematopoietic cell expansion through the Notch pathway [26], the dynamics of expression of Notch and their ligands (Jagged2 and DLLA4) were evaluated in co-culture. An increase in Notch1 and Jagged2 was found in adherent hematopoietic cells and EC after 72 h of co-culture. This could suggest that this axis may be involved in the G0 cell cycle arrest observed in the leukemic population. Similar results were reported using primary samples and CML cell lines, in which the overexpression and Notch1 activity was involved in the quiescence of leukemic stem cells, conferring them protection [27]. The Notch1–Jagged2 signal axis, in conjunction with BCR::ABL1 expression, has been implicated in CML progression to blast crisis [16]. Thus, functional studies are needed to corroborate the role of such elements in the quiescence of CML stem cells.

Our study shows, for the first time, an in vitro, co-culture system that seems adequate to evaluate the effects of the endothelial microenvironment on primitive leukemic cells. Since ECFC is the only adult cell population reported to date that possesses both angiogenic and repair capacities, and considering that these cells are usually modified when in contact with cells from different diseases, this experimental system may be relevant to study biological responses in different physiological states and disorders [28].

The results of our study show that co-culture between primitive CML cells and normal endothelial cells, without any additional factor, generates a microenvironment that allows for the in vitro maintenance of primitive CML cells through their permanence in a quiescent state, their proliferation and differentiation, which seem to be associated with the generation of an inflammatory environment, and their own adhesion abilities that could involve the Notch1–Jagged2 axis. In this context, it is necessary to evaluate these same biological effects using endothelial cells derived from CML bone marrow, in which the observed effects can be greater since endothelial cells may already possess functional alterations.

## 4. Materials and Methods

### 4.1. Patient Samples

Bone marrow (BM) cells were collected from seven CML patients in Chronic Phase (CP) at the time of diagnosis, ranging in age from 39 to 71 years. All patients fulfilled the standard criteria for CML, including the presence of the Ph chromosome translocation in direct marrow preparations. The procedures were performed at the Hematology Department, Medical Specialties Hospital, La Raza Medical Center, IMSS, Mexico City. Normal bone marrow (NBM) samples were obtained from five hematological normal adults, ranging in age from 47 to 81 years, subject to hip replacement surgery at Villa Coapa General Regional Hospital, IMSS, Mexico City. All of these procedures were approved by the Scientific and Ethics Committee of National Medical Center, IMSS (Register R-2012-785-084). In all cases, written informed consent was obtained from each one of the donors.

### 4.2. Lin- CD34+ Cell Enrichment

Mononuclear cells (MNC) were isolated using a Lymphoprep^TM^ (Stem Cell Technologies Inc., Vancouver, Canada)-based cell gradient. This cell fraction was re-suspended in phosphate-buffered saline (PBS) solution (Biowest) supplemented with 3% fetal bovine serum (Gibco) to proceed to enrichment of Lin-CD34+ cells by negative selection, according to the technical guidelines of StemSep^TM^ system (StemCell Technologies Inc., Vancouver, BC, Canada). Briefly, MNC were incubated for 15 min with the antibody cocktail (CD2, CD3, CD14, CD16, CD19, CD24, CD56, CD66b and glycophorin A), followed by incubation with magnetic colloid. The Lin-CD34+ cells were collected in basal medium EBM-2^TM^ SingleQuots with 100 U/mL penicillin and 100 μg/mL streptomycin.

In all cases the enrichment percentage of normal and leukemic CD34+lin- cells was greater than 60%. It is important to clarify that since this cell population contains both stem (CD34+CD38-lin-) and progenitor (CD34+CD38+lin-) cells, it will be considered as a primitive population.

### 4.3. Endothelial Cells

Human umbilical vein endothelial cells (HUVEC) and endothelial colony forming cells (ECFC) were purified from healthy newborns and peripheral blood from healthy donors, respectively, and were identified by the presence of CD31, CD146 and CD309, and the absence of CD45, CD90 and CD14, as previously described [29]. Early passage (<5) of ECFC or HUVEC were seeded in 12-well culture plates pre-coated with type 1 rat tail collagen (BD Biosciences, Bedford, MA, USA) at 100,000 cells/cm^2^, in a humidified incubator at 37 °C and 5% CO_2_. Endothelial cell cultures were maintained with EGM-2^TM^ SingleQuots medium (Lonza, Walkersville, MD, USA) fully supplemented and it was changed every three days. The endothelial cell layer was washed three times with PBS (1X) before each co-culture.

### 4.4. MEG-01 Cell Line

MEG-01 (ATCC) is a megakaryoblast leukemia cell line derived from CML in blast crisis. MEG-01 was maintained at a density between 1.5 × 10^5^–1 × 10^6^ cells/mL in RPMI 1640 (Gibco) at 10% of fetal bovine serum (FBS), 100 U/mL penicillin and 100 μg/mL streptomycin. The culture medium was changed every three days. The cells were washed twice with PBS (1X) before co-culture.

### 4.5. Co-Culture of Hematopoietic Cells with Endothelial Cells

For the co-cultures with contact, primitive hematopoietic cells (Lin-CD34+) were plated at 1 × 10^5^ cells/cm^2^ over the endothelial cell monolayers at a confluence of 100%. In co-cultures without contact, Lin-CD34+ cells at the same density, were seeded in Costar Transwell (Corning Costar, Tewksbury, MA, USA) 12 mm diameter inserts with polyester membrane and 0.4 µm pore size. These inserts were layered over endothelial cell monolayers. Contact co-cultures with the MEG-01 cell line were performed at ≤5 passage seeded over HUVEC monolayer. All co-cultures were performed at a 1:1 ratio between EC and hematopoietic cells and were maintained at 37 °C in 5% CO_2_ for three days in a humidified incubator in 1.5 mL/cm^2^ of EBM-2 basal medium (Lonza) with 100 U/mL penicillin and 100 μg/mL streptomycin, without supplements or fetal bovine serum (FBS). For negative controls, Lin-CD34+ or MEG-01 cells were cultured with the same basal medium, and for quality control culture, Lin-CD34+ cells were maintained using StemSpan^TM^ (Stem technologies) medium supplemented with 10 ng/mL of TPO, IL-3, IL-6, SCF, FLT3-L, GM-CSF and G-CSF.

### 4.6. Hematopoietic Cells’ Recovery

After three days or every 24 h of co-culture, total hematopoietic cells from two distinct cell fractions were collected separately. Briefly, the supernatant (non-adherent hematopoietic fraction) was collected and the cell layer that remained attached was gently washed twice with PBS with Ca^2+^ and Mg^−^ to remove the remaining non-adherent cells that were also collected in the same fraction. The adherent fraction was treated with TrypLE^TM^ Express enzyme during 2–3 min at 37 °C; after this time, all adherent cells (hematopoietic and endothelial) were collected. Both cell fractions were counted with Trypan blue (viability > 90%) and incubated with specific antibodies for the identification between hematopoietic (CD45+) and endothelial (CD31+ or CD146+) cells, as described in flow cytometry analysis.

### 4.7. Immunophenotype and Adhesion Molecules

Primitive immunophenotype was analyzed by immune-label with anti-lineage-FITC, antiCD34-PECy7, antiCD38-APC and antiCD26-PE markers. For myeloid immunophenotype, cells were stained with antiCD14-PE. The presence of Notch1 and their ligands was determined by staining hematopoietic and endothelial cells with anti-Notch1-APC, anti-DLL-4-PE and anti-Jagged2-PE. All incubations were performed in the dark. Hematopoietic cells were gated according to their forward and side scatter properties and CD45 presence and CD31 or CD146 absence.

### 4.8. Colony Forming Cell Assays

The evaluations of colony forming cells (CFC) were conducted after three days in co-culture with endothelial cells. Briefly, 3 × 10^3^ hematopoietic cells from different co-cultures, were sub-cultured for 14 days in complete methylcellulose medium with recombinant cytokines (MethoCult^TM^ H4434, StemCell Technologies Inc., Vancouver, BC, Canada). CFC were defined as clusters consisting of 40 or more cells and were quantified using an inverted microscope (CKX41SF, Olympus Corporation, Tokyo Japan) under 4X and 10X. The counting of CFC was normalized with the initial number of CFC at time zero and after enrichment, of each sample.

### 4.9. Proliferation and Cell Cycle Assay

Division index was measured using carboxyfluorescein succinimidyl ester (CFSE) staining. Lin-CD34+ or MEG-01 cells were labeled by CFSE according to the manufacturer’s instructions and culture with or without EC as described above. At 24, 48 and 72 h, adherent and non-adherent co-cultured cells and control hematopoietic cells were analyzed using flow cytometry (FACSVerse; BD). The division index was quantified according to CFSE signal intensity using FlowJo software.

The CD34+CD38+ and CD34+CD38- populations cells were analyzed throughout time of culture using antiCD31-PB, antiCD34-PECy7, antiCD38-APC and CFSE multi-staining. The cell cycle assay was performed as described previously [30]. Briefly, cells were fixed with cold paraformaldehyde at 4% for 20 min at 4 °C. They were washed twice with staining buffer (PBS with 3% of FBS) and the cell membrane staining performed. The cells were incubated with antiCD34-PECy7, antiCD38-APC and antiCD31-PB for 30 min at room temperature, and were incubated with permeabilization solution (0.1% Triton X-100, 10% FBS in 1X PBS) for 15 min at room temperature. Cells were washed and incubated with antiKi67-FITC for 30 min and with DAPI (2 μM) for 10 min at room temperature to finally be analyzed by multiparametric flow cytometry (FACSVerse; BD).

### 4.10. Cytokines’ Analysis

Supernatants from co-cultures and control cultures were recovered, centrifuged at 2000 rpm for 5 min and stored at −70 C until cytokines’ evaluation. Tests were performed on 25 μL samples using multiplex immunoassay MILLIPLEX MAP Human Cytokine/Chemokine Magnetic Bead (Merck Millipore, Billerica, MA) and LegendPlex multi-analyte Flow assay kit (Biolegend Inc). Cytokines analyzed were: vascular-endothelial growth factor A (VEGF-A), FLT-3-ligand, granulocyte-macrophage colony stimulating factor (GM-CSF), epidermal growth factor (EGF), interleukin 3 (IL-3), interleukin 6 (IL-6), interleukin 8 (IL-8), tumor necrosis factor α (TNF-α), monocyte inhibitor protein 1 α (MIP-1α), monocyte inhibitor protein 1 β (MIP-1β), interferon gamma (IFNγ), transforming growth factor β (TGFβ), interleukin 1α (IL-1α), fibroblast growth factor 2 (FGF-2), granulocyte colony stimulating factor (G-CSF), stromal cell-derived factor 1 α (SDF-1α/CXCL12), monocyte chemo attractant protein-1 (MCP-1/CCL2), leukemia inhibitory factor (LIF), stem cell factor (SCF) and placental growth factor (PlGF). The data were reported in pg/mL using MagPix and MILLIPLEX Software Analyzer or in FACS Canto (BD) and LegendPlex Software.

### 4.11. Statistical Analysis

Two-way ANOVA and Tukey test were used to analyze results between groups. For comparisons with the control group, Dunnett’s test was used. *p*-value < 0.05 was considered statistically significant. Statistical analysis was performed using GraphPad Prism software, version 6.0c (GraphPad Software, San Diego, CA, USA). Results are shown as the mean ± SEM, or only mean in some cases, of triplicate assays.

## Supplementary Materials

The following supporting information can be downloaded at: https://www.mdpi.com/article/10.3390/ijms231810326/s1.
