# Supplementary material for: Cell Contact with Endothelial Cells Favors the In Vitro Maintenance of Human Chronic Myeloid Leukemia Stem and Progenitor Cells
Source: Int J Mol Sci. 2022 Sep 7;23(18):10326. doi: 10.3390/ijms231810326 (PMC9499491; doi:10.3390/ijms231810326)
Supplement: Supplementary file 1 [file ijms-23-10326-s001.zip › ijms-1765317-supplementary.pdf]

SUPPLEMENTARY MATERIALS

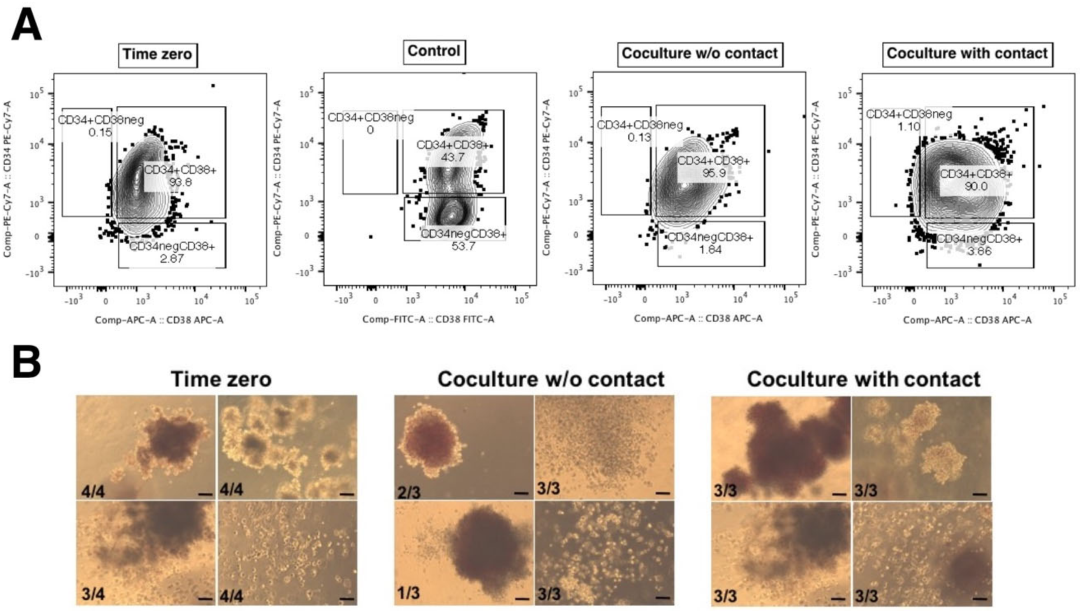

Supplementary Figure S1.

(A) Representative dot plots of CML lin-CD34+ immunophenotype evaluation after three days of culture in basal medium (control), or in co-culture with or without direct contact with Endothelial Cells. The gates show the percentage of each analyzed subpopulation. (B) After each co-culture, representative photographs of colony forming cells (CFC) assay found. The numbers indicate the frequency of each kind of colonies found in different analyzed samples.

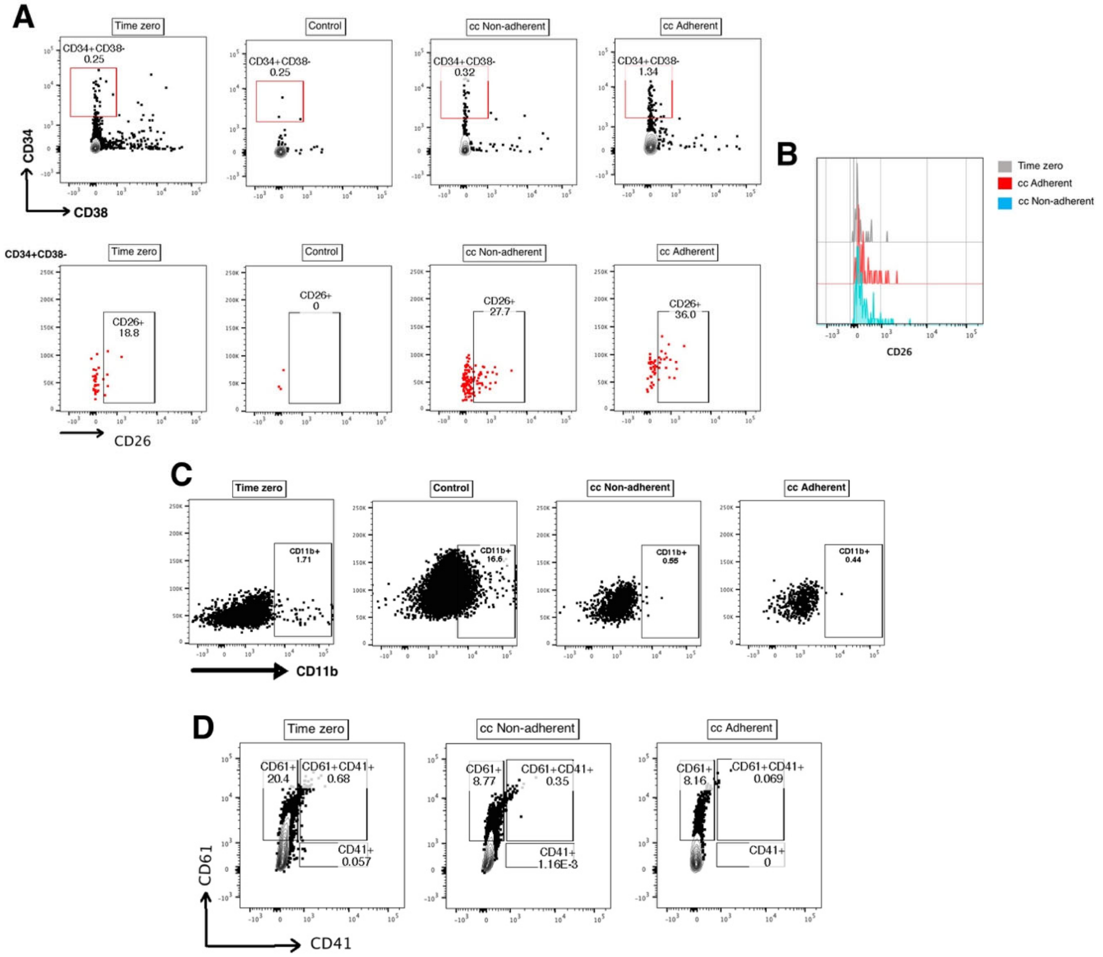

Supplementary Figure S2.

(A) Representative dot plot of stem cell immunophenotype of MEG-01 at zero time and after three days of EC-co-culture or with basal medium (Control). The adherent and the non-adherent fraction of the same co-cultures were analyzed separately. Stem cells: $CD34^{+}CD38^{neg}CD26^{+}$. (B) Mean intensity of fluorescence (MIF) of CD26 in MEG-01 after co-culture with EC. (C) Representative dot plot of myeloid CD11b+ cells in CML samples after three days of co-culture. (D) Representative DotPlot of CD61+CD41+ cells in MEG-01 cell line after three days of co-culture.

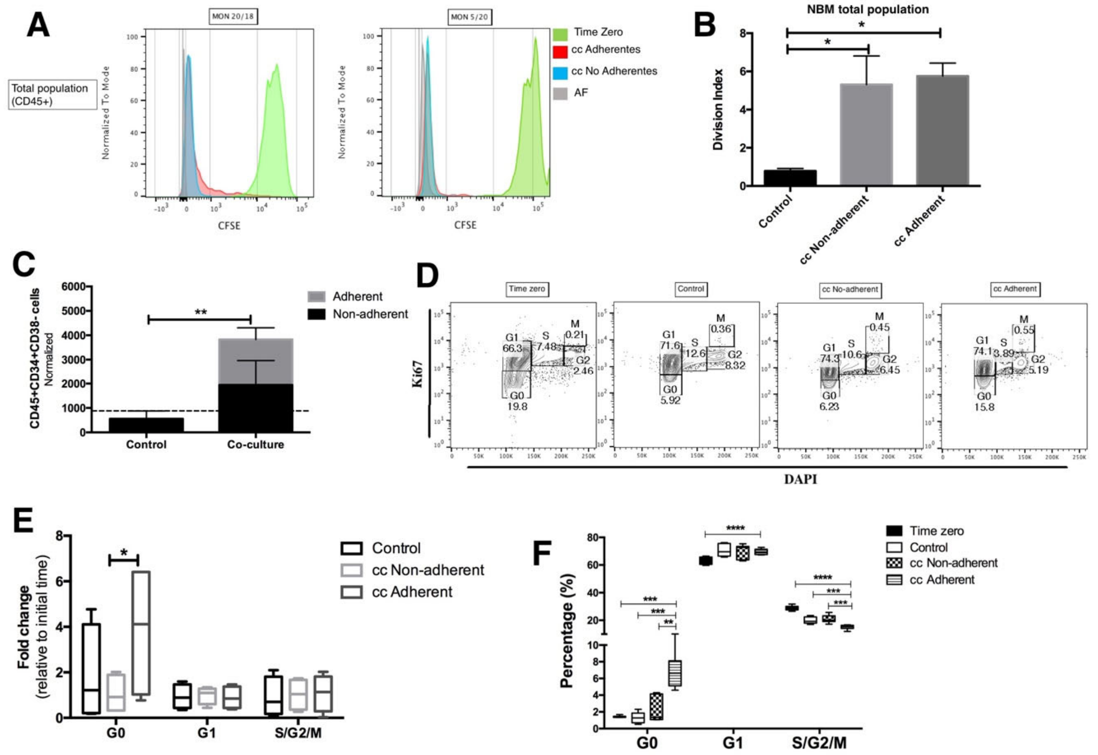

Supplementary Figure S3.

The proliferation of CD34+ from Normal Bone Marrow Cells after three days of co-culture in contact with EC. (A) Representative histograms of CFSE assay in adherent and non adherent fraction to EC from the normal total population. (B) Cell division index in adherent and non adherent fraction of normal total population. (C) The number of stem cell (Lin-CD34+CD38-) in the MEG-01 cell line co-cultured with EC for three days. The number was normalized with the initial number of stem cells found at the beginning of each assay. (D) Representative dot plots of cell cycle status of one Normal Bone Marrow sample after three days in co-culture with EC. (E) Fold change of cell cycle phases, relative to time zero of each sample used. (F) Cell cycle status of the MEG-01 cell line after three days of co-culture.
